# Normal cerebrospinal fluid protein and associated clinical characteristics in children with tuberculous meningitis

**DOI:** 10.1080/07853890.2021.1937692

**Published:** 2021-06-14

**Authors:** Jun-Li Wang, Chao Han, Feng-Lian Yang, Mao-Shui Wang, Yu He

**Affiliations:** a Department of Lab Medicine, The Affiliated Hospital of Youjiang Medical University for Nationalities, Baise, China; b Department of Geriatrics, Shandong Mental Health Center, Jinan, China; c School of Pharmacy, Youjiang Medical University for Nationalities, Baise, China; d Department of Lab Medicine, Shandong Provincial Chest Hospital, Cheeloo College of Medicine, Shandong University, Jinan, China; e Department of Clinical Laboratory, First Affiliated Hospital of Guangxi Medical University, Nanning, China

**Keywords:** Children, tuberculous meningitis, cerebrospinal fluid protein, risk factor

## Abstract

**Background:**

Although abnormal cerebrospinal fluid (CSF) protein can be used to predict the outcome of tuberculous meningitis (TBM) and diagnose TBM, normal CSF protein remains a concern in patients with TBM. This retrospective study aimed to assess the clinical characteristics associated with normal CSF protein, to resolve the dilemma of CSF protein in the management of childhood TBM.

**Methods:**

Between January 2006 and December 2019, consecutive child patients (≤15 years old, a diagnosis of TBM, and tested for CSF protein) were included for analysis. CSF protein was tested on a chemistry analyzer using the pyrogallol red-molybdate method. Abnormal CSF protein was defined as >450 mg/L. Patient characteristics were collected from the electronic medical records. Then, characteristics associated with normal CSF protein were estimated in the study, using univariate and multivariate logistic regression analysis.

**Results:**

A total of 125 children who met the criteria were enrolled during the study period. Twenty-nine patients had a normal CSF protein and 96 had an abnormal CSF protein. Multivariate analysis (Hosmer–Lemeshow goodness-of-fit test: χ^2^=2.486, df = 8, *p* = .962) revealed that vomiting (age- and sex-adjusted OR = 0.253, 95% CI: 0.091, 0.701; *p* = .008) and serum glucose (>5.08 mmol/L; age- and sex-adjusted OR = 0.119, 95% CI: 0.032, 0.443; *p* = .002) were associated with the normal CSF protein in childhood TBM.

**Conclusion:**

In suspected childhood TBM, patients without vomiting or having low serum glucose are easy to present with normal CSF protein. Hence, when interpreting the level of CSF protein in children with such characteristics, a careful clinical assessment is required.

**KEY MESSAGES**
- In suspected childhood tuberculous meningitis, patients without vomiting or having low serum glucose are easy to present with normal CSF protein. Hence, when interpreting the level of CSF protein in children with such characteristics, a careful clinical assessment is required.

## Introduction

Currently, tuberculosis (TB) remains one of the most important infectious diseases for children. According to a WHO report (2018), it was estimated that 10 million people fell ill with TB and 1.5 million deaths occurred due to TB, children accounted for 11% of all TB cases, and 14% of cases that died from TB were children [1]. Tuberculous meningitis (TBM) is thought of as one of the serious forms of TB disease. It was reported that 15%-30% of children with TBM died [2–4], and survivors are easy to have neurological disabilities [5,6]. Therefore, timely diagnosis and appropriate treatment are required to improve the current dilemma of the disease.

The diagnosis of TBM remains a difficult, especially in children. This is because that when cerebrospinal fluid is employed, routine microbiological methods such as acid-fast bacilli (AFB) smear, polymerase chain reaction (PCR), and culture usually have a low sensitivity for the diagnosis of TBM [7]. Moreover, childhood TB is a paucibacillary illness. Hence, the diagnostic assays are not considered as the only criteria for the diagnosis. Recently, a case definition for TBM has been addressed, including several aspects such as presentation, CSF findings, imaging, and TB evidence elsewhere.

Abnormal CSF protein is an important clinical presentation for TBM patients. Moreover, CSF protein can be used to predict the outcome of TBM and served as a tool for the diagnosis of TBM [8–11]. Unfortunately, normal CSF protein remains a concern in patients with TBM [12]. Remarkably, normal CSF protein may lower the suspicion of TBM in children. Therefore, investigating the clinical characteristics associated with normal CSF protein is essential for the management of childhood TBM.

## Methods

The study protocol was approved by the Ethics Committee of Shandong Provincial Chest Hospital, Jinan, China (NO. 2020XKYYEC-29) and the retrospective study was conducted in compliance with the Helsinki declaration. Due to its retrospective nature and anonymous data collection, written informed consent was exempted by the Ethics Committee.

Between January 2006 and December 2019, consecutive child patients aged ≤15 years old, had a diagnosis of TBM, and were tested for CSF protein were included for the analysis. TBM was defined if one of the following criteria (modified from the criteria established by Marais S et al. [13]) was met: 1) definite: acid-fast bacilli (AFB, +) on cerebrospinal fluid (CSF) microscopy, or CSF TB- PCR(+), or *M.tuberculosis* cultured from CSF. 2) conclusive: symptoms and signs of meningitis and abnormal CSF findings (such as total white cell count >5 cells × 10^6^/L, protein >0.45 g/L, glucose <2.2 mmol/L, and CSF/serum glucose ratio <0.5), *plus* at least one of the following i) TB suggested by abnormal radiographic features (chest, or cerebral imaging), ii) positive TB assays (such as AFB, PCR, and culture) using non-CSF samples.

CSF protein was tested on ADVIA 2400 chemistry analyzer (Siemens, IL, USA) using a commercial kit (Pyrogallol red-molybdate method, LEADMAN, Beijing, China). Abnormal CSF protein was defined as >450 mg/L. Patient data, such as demographic, clinical, laboratory, and radiographic features were collected from the electronic medical records. Subsequently, the characteristics associated with normal CSF protein were then estimated in the study.

Statistical analyses were performed using SPSS version 16.0 (SPSS, Chicago, IL, USA). Continuous variables were expressed as mean ± standard deviation (SD) and categorical variables as count (percentages). Univariate and multivariate logistic regression analyses adjusted by age and sex were performed to determine characteristics associated with normal CSF protein, and odds ratios (OR) and corresponding 95% confidence interval (CI) were also calculated [14]. Additionally, to make a better clinical understanding, continuous variables were transformed into categorical variables, based on cut-offs determined by the receiver operating characteristic curve (ROC) analysis. The goodness-of-fit was assessed using the Hosmer–Lemeshow test. A *p* value <.05 was considered significant.

## Results

### Patient characteristics

A total of 125 children meeting the criteria was enrolled during the study period. Twenty-nine patients (23.2%) had a normal CSF protein and 96 (76.8%) had an abnormal CSF protein. Table 1 shows a comparison of clinical-pathological characteristics between patients with normal and abnormal CSF protein.

**Table 1.** Univariate analysis of the demographic data associated with normal CSF protein in childhood TBM.

	Total (*n*)	Normal CSF protien group (*n*)	Abnormal CSF protien group (*n*)	*p* value	OR (95% CI)
N	125	29 (23.2%)	96 (76.8%)		
CSF protein (mg/L)	984 ± 758	307 ± 100	1189 ± 752		
Symptoms					
Cough	27 (21.6%)	7 (24.1%)	20 (20.8%)	.705	
Fever	112 (89.6%)	27 (93.1%)	85 (88.5%)	.485	
Vomitting	61 (48.8%)	7 (24.1%)	54 (56.3%)	.004	0.247 (0.097, 0.634)
Headache	65 (52.0%)	13 (44.8%)	52 (54.2%)	.379	
Coma	16 (12.8%)	4 (13.8%)	12 (12.5%)	.855	
Drowsiness	11 (8.8%)	0 (0.0%)	11 (11.5%)	.999	
Convulsion	20 (16.0%)	3 (10.3%)	17 (17.7%)	.349	
Dizziness	5 (4.0%)	1 (3.4%)	4 (4.2%)	.863	
Clinical Chemistry (serum)					
Total protein (g/L)	69 ± 8	67 ± 6	69 ± 8	.173	
Albumin (g/L)	40 ± 5	39 ± 5	41 ± 4	.036	0.908 (0.830, 0.994)
Blood urea nitrogen (mmol/L)	4.5 ± 3.2	4.2 ± 1.2	4.6 ± 3.6	.621	
Creatinine (μmmol/L)	37.6 ± 16.1	41.3 ± 14.6	36.5 ± 16.4	.164	
Glucose (mmol/L)	5.0 ± 1.2	4.5 ± 0.9	5.2 ± 1.2	.005	0.452 (0.260, 0.785)
Lactate dehydrogenase (U/L)	279 ± 276	215 ± 56	293 ± 302	.045	0.989 (0.979, 1.000)

Abbreviations: CSF: cerebrospinal fluid; TBM: tuberculous meningitis; OR: odds ratio; CI: confidence interval.

The mean age and weight were 7.6 ± 5.2 years and 24 ± 16 Kg, respectively. Of the 125 child patients, 66 patients (52.8%) were male and 102 (81.6%) were from rural areas. Additionally, other sites involved with TB were reported as pulmonary (*n* = 59, 47.2%), milliary (*n* = 22, 17.6%), pleural (*n* = 5, 4.0%), and lymph node (*n* = 3, 2.4%).

The frequencies of hospitalization were 2.2 ± 1.8, and the mean treatment delay was reported at 33 ± 35 d. Among the enrolled patients, 29 patients (23.2%) had a TB contact history and 109 patients (87.2%) were transferred from a teaching hospital. Almost all patients (*n* = 120, 96.0%) had inpatient therapy, over half (*n* = 75, 60.0%) had outpatient therapy, and a significant proportion (*n* = 16, 12.8%) were reported having self-treatment. In addition, a majority (*n* = 99, 79.2%) of patients experienced antibiotics therapy and a few (*n* = 22, 17.6%) were administrated with anti-TB therapy

The vital signs were as follows: temperature, 37.3 ± 0.9 °C; heart rate, 100 ± 22 beats/min; respiratory rate, 22.7 ± 3.0 breaths/min; systolic pressure, 107 ± 16 mmHg; diastolic pressure, 69 ± 11 mmHg. The symptoms were as follows: fever (*n* = 112, 89.6%), headache (*n* = 65, 52.0%), vomiting (*n* = 61, 48.8%), cough (*n* = 27, 21.6%), convulsion (*n* = 20, 16.0%), coma (*n* = 16, 12.8%), drowsiness (*n* = 11, 8.8%), and dizziness (*n* = 5, 4.0%). CSF analysis is shown in Table 2.

**Table 2.** The CSF findings in children with TBM.

	Total (*n*)
*N*	125
Protein (mg/L)	984 ± 758
White blood cell (10^6^/L)	159 ± 288
Mononuclear cell (%)	77 ± 25
Polyonuclear cell (%)	24 ± 25
Lactate (mmol/L)	4.6 ± 2.0
Aspartate aminotransferase (U/L)	15 ± 11
Total bilirubin (mmol/L)	1.01 ± 2.57
Adenosine deaminase (U/L)	5.5 ± 10.8
Lactate dehydrogenase (U/L)	69 ± 103
α- hydroxybutyrate dehydrogenase (U/L)	45 ± 55
Total cholesterol (mmol/L)	0.15 ± 0.44
Glucose (mmol/L)	2.01 ± 1.07
IgA (mg/L)	18.4 ± 13.5
IgG (mg/L)	80.6 ± 31.5
IgM (mg/L)	4.3 ± 1.2
Chloride (mmol/L)	113 ± 7

Abbreviations: CSF: cerebrospinal fluid; TBM: tuberculous meningitis.

T-SPOT.TB was tested in 37 patients and 26 (70.3%) of them had a positive result. Other lab examinations such as blood cell count and flow cytometry, were shown in Table 1 and Supplementary Table 1. Additionally, CSF analysis is shown in Table 2.

### Univariate and multivariate analysis

Table 1 shows the results of univariate analysis between normal and abnormal CSF protein groups. It was found that normal CSF protein was associated with vomiting (OR = 0.247, 95% CI: 0.097, 0.634; *p* = .004), serum albumin (OR = 0.908, 95% CI: 0.830, 0.994; *p* = .036), serum glucose (OR = 0.452, 95% CI: 0.260, 0.785; *p* = .005), and serum LDH (OR = 0.989, 95% CI:0.979, 1.000; *p* = .045).

Further multivariate analysis (Hosmer–Lemeshow goodness-of-fit test: *χ*^2^=2.486, df = 8, *p* = .962) revealed that vomiting (age- and sex-adjusted OR = 0.253, 95% CI: 0.091, 0.701; *p* = .008) and serum glucose (>5.08 mmol/L; age- and sex-adjusted OR = 0.119, 95% CI: 0.032, 0.443; *p* = .002) were associated with the normal CSF protein in childhood TBM (Table 3).

**Table 3.** Age- and sex-adjusted OR for risk factors associated with normal CSF protein in childhood TMB.

	Adjusted (age and sex) OR	*p* value
Vomitting	0.253 (0.091, 0.701)	.008
Serum glucose (>5.08mmol/L)	0.119 (0.032, 0.443)	.002

Abbreviations: CSF: cerebrospinal fluid; TBM: tuberculous meningitis; OR: odds ratio; CI: confidence interval.

## Discussion

It was well characterized that elevated CSF protein levels were associated with a poor outcome in TBM. For example, raised CSF protein was recently found to be a significant predictor of blindness [15]. Therefore, assessing the CSF level of total protein would aid to improve the management of TBM in children. Nevertheless, in some TBM cases, normal CSF protein remains a concern [16,17]. Interestingly, according to our results, normal CSF protein was associated with clinical presentations such as without vomiting and low serum glucose. Therefore, these characteristics suggested by our findings may improve the current knowledge of the role of CSF protein in the management of childhood TBM and improve the dilemma in the diagnosis of childhood TBM.

Increased level of CSF protein is known as one of the main presentations of TBM. CSF protein is composed of albumin, immunoglobulin, and transferrin, along with other enzymes and globulins. In a previous study, the onset of TBM was characterized by a rise in the alpha-1 and gamma globulin fractions and by a decrease in the beta and pre-albumin fractions. In general, the abnormal CSF protein is widely explained by brain damage, which is related to the severity of meningeal inflammation and blood-brain barrier dysfunction [18,19]. Serial CSF examination demonstrated that CSF protein concentration changes slowly during the treatment of TBM [20], and if appropriate treatment is administrated, unlike a rapid decrease in neutrophil count and CSF glucose, CSF protein showed a steady decline gradually [20]. Nevertheless, alpha 1 fraction and pre-albumin tend to normalize after initiation of anti-TB therapy [21]. Besides the above-mentioned, low CSF protein levels also occur in some conditions such as repeated lumbar puncture, a chronic leak, infant, and acute water intoxication [22].

Our data suggested that vomiting was associated with an abnormal CSF protein. Therefore, in suspected children without vomiting, CSF protein may be prone to present at a normal state. Previously, a similar finding has been reported in consistency with our findings. Usually, vomiting as a presenting symptom is associated with cranial nerve involvement [23]. Sharma P et al. reported that elevated levels of CSF protein and cells are predictors for cranial nerve involvement [9]. This may be explained that high levels of CSF protein and cell count may cause more formation of basal exudates, leading to cranial nerve involvement. Furthermore, the presence of vomiting was associated with poor outcomes of TBM [24]. This is possible because patients with vomiting represent a more severe form of the disease.

Our study demonstrated that elevated serum level of glucose is associated with abnormal CSF protein. This means that a low level of glucose occurs more commonly in patients with normal CSF protein. Identifying this characteristic is easy and would help to interpret the normal CSF protein in childhood TBM. The association between serum glucose and CSF protein may be explained by the influence of diabetes on the CSF level of protein. First, diabetes mellitus is taken as a factor of the diagnostic scoring system for TBM diagnosis [25]. Second, in TBM, diabetes mellitus is more common in patients with ischaemic zone infarction than those without [26,27]. Third, as a variable in the scoring and cumulative score systems, diabetes mellitus is used to predict the unfavourable outcome of TBM and provide a linear estimation of prognosis [28]. Interestingly, similar findings were reported in CSF protein among TBM patients. Although the cut-off value (5.08 mm/L) defined in the study is lower than the criteria for diabetes mellitus, it may be more practical in clinical practice. Remarkably, if a patient with a low level of serum glucose (≤5.08 mm/L) was admitted for suspected TBM, a high risk of normal CSF protein may be presented.

Although the study has several interesting findings, some limitations are still to be considered. First, this was a single centre experience, it thus may only reflect a local epidemiological situation. Second, the study had a retrospective nature, sample size and selection bias should be taken into account. Third, the criteria for abnormal CSF protein in TBM varied widely, this may have a significant impact on the result. Fourth, associations between CSF protein and other CSF analyses were not investigated, due to its close correlations with clinical course. Therefore, further studies are needed to determine the reference of CSF protein for the diagnosis of TBM, and a large prospective study is required in the future to validate the above findings.

## Conclusions

Our study found that, in suspected childhood TBM, patients without vomiting or having low serum glucose are easy to present with a normal CSF protein. A normal CSF protein may lower the suspicion of TBM in children and result in delayed diagnosis and anti-TB treatment. Therefore, when interpreting the level of CSF protein in children with such characteristics, a careful clinical assessment is required.

## Data Availability

The data that support the findings of this study are available from the corresponding author, [MSW or YH], upon reasonable request.
